# One-splint versus two-splint technique in orthognathic surgery for class III asymmetry: comparison of patient-centred outcomes

**DOI:** 10.1007/s00784-021-03967-9

**Published:** 2021-05-05

**Authors:** Jing Hao Ng, Ying-An Chen, Yuh-Jia Hsieh, Chuan-Fong Yao, Yu-Fang Liao, Yu-Ray Chen

**Affiliations:** 1 Department of Orthodontics, National Dental Centre Singapore, Singapore, Singapore; 2 Craniofacial Center, Chang Gung Memorial Hospital, Taoyuan, Taiwan; 3 Craniofacial Research Center, Chang Gung Memorial Hospital, Linkou, Taiwan; 4 Department of Plastic and Reconstructive Surgery, Chang Gung Memorial Hospital, Linkou, Taiwan; 5 Department of Craniofacial Orthodontics, Chang Gung Memorial Hospital, Taoyuan, Taiwan; 6 College of Medicine, Chang Gung University, Taoyuan, Taiwan

**Keywords:** Quality of life, Clinical outcomes, Comparative effectiveness research (CER), Facial symmetry, Orthognathic surgery, One-splint

## Abstract

**Objectives:**

Two-jaw orthognathic surgery (OGS) is done using either the one-splint technique with free-hand positioning of the maxillomandibular complex or the two-splint technique with intermediate splints to position the maxilla. It is uncertain which technique achieves better outcomes. This study compares frontal soft tissue symmetry and subjective patient QoL between one-splint and two-splint techniques in skeletal Class III asymmetry patients undergoing OGS with three-dimensional surgical planning.

**Materials and methods:**

This retrospective case-control study comprised 34 one-splint and 46 two-splint OGS patients. Frontal photographs and Orthognathic Quality of Life Questionnaire (OQLQ) were done pre- and post-treatment. Frontal soft tissue symmetry was analysed with the anthropometric Facial Symmetry Index. Measurements were compared with *t*-tests and chi-squared tests with *p*-value set at 0.05.

**Results:**

The groups differed in pre-treatment ANB and OQLQ scores. The two-splint group showed significant improvement in all symmetry measures. The one-splint group showed significant improvement in all symmetry measures except midface deviation, upper contour deviation and the Facial Contour Symmetry Index. Both groups showed significant improvement in OQLQ scores. There were no significant differences in post-treatment symmetry measurements and OQLQ scores between groups.

**Conclusions:**

Although two-splint technique may better improve contour symmetry, there were no significant differences in frontal soft tissue symmetry and QoL after OGS in skeletal Class III asymmetry with either one-splint or two-splint technique, with both techniques resulting in significant improvement.

**Clinical relevance:**

One-splint and two-splint surgical techniques produce similar patient-centred outcomes in Class III asymmetry patients.

**Supplementary Information:**

The online version contains supplementary material available at 10.1007/s00784-021-03967-9.

## Introduction

Patients with facial asymmetry frequently undergo bimaxillary orthognathic surgery (OGS) for improvement of dentofacial symmetry and aesthetics [1–4]. Two-jaw OGS can be carried out either using the one-splint technique with free-hand positioning of the maxillomandibular complex (MMC) [5–7] or the two-splint technique with intermediate splints to position the maxilla [8].

Prior to 2003, the authors’ Craniofacial Center used the classic two-splint OGS technique with two-dimensional (2D) surgical planning with mounted plaster models and lateral and postero-anterior cephalograms. After 2003, due to less than ideal results with the two-splint technique, a one-splint technique using only the final occlusal splint with free-hand positioning of the MMC was developed [6, 7]. Mild under- or over-correction of hard tissue asymmetry was used to achieve soft tissue symmetry, with intra-operative assessment needed due to patient variability in soft tissue response [6, 7, 9]. This was validated by Kim et al. [10], who found that the average amount of lip cant correction was approximately 50% of the maxillary occlusal cant correction. With 2D planning, the one-splint technique produces good results in asymmetry patients [6, 11]. It also reduces technical effort because only one splint is fabricated. However, whilst diagnosis, surgical planning and treatment outcomes are assessed with the patient upright and with relaxed soft tissues, the one-splint technique relies on supine intra-operative assessment of the patient with soft tissues that are invariably distorted by airway intubation. This introduces added complexity to the one-splint technique. The technique is hence surgically demanding, has a steep learning curve and depends heavily on surgeon experience [7, 12, 13].

In 2015, the Center started virtual orthognathic planning using three-dimensional (3D) software, cone beam computed tomography (CBCT), photographs and digital dental casts. Using 3D planning with one-splint technique achieves good hard and soft tissue results in asymmetry patients [13–16] and seems to outperform 2D surgical planning with one-splint technique in terms of hard tissue gonial symmetry and yaw symmetry achieved [14] as well as improvement in soft tissue facial midline asymmetry [16].

However, even when the 3D simulation and a detailed 3D surgical plan are provided, the MMC movement is greatly dependent on the surgeon’s intra-operative assessment of the checkpoints when using the one-splint technique [13, 14]. The one-splint technique produces significant deviation from the 3D surgical plan, with a relatively poor landmark accuracy of 1.5 mm at the maxilla and 2 mm at the mandible, with MMC pitch showing the greatest error [13]. From 2015, due to the desire to translate the precise 3D-planned skeletal movements to the surgery, the surgical team shifted from one-splint to two-splint technique with a 3D printed intermediate splint. This required greater technical effort to produce two surgical splints, as well as reduced intra-operative flexibility in changing the surgical planning. The benefit of the two-splint technique is having surgical planning done in the upright patient being congruently translated into the intra-operative and post-operative condition, rather than depending on a supine intra-operative condition to decide the MMC position as in the one-splint technique [13, 14, 17, 18].

From 2015 to 2016, the Center principally used the one-splint technique with 3D planning and, from 2017 onwards, predominantly used the two-splint technique with 3D planning. It is uncertain which technique achieves better outcomes. This study aims to compare frontal soft tissue symmetry and subjective patient-reported health-related quality of life (QoL) between one-splint and two-splint techniques in skeletal Class III asymmetry patients undergoing OGS with 3D surgical planning.

## Methods

### Subjects

This retrospective case-control study comprised patients identified from the Center’s database. All patients meeting the following selection criteria were included in the study: at least 18 years of age with stable body height, skeletal Class III with ANB angle ≤ 0 degree, facial asymmetry with significant menton deviation ≥ 4 mm as assessed on a frontal cephalogram [19–21] or significant contour asymmetry assessed by an orthodontist (L.Y-F) on frontal photos, had bimaxillary OGS from year 2015 to 2019 with LeFort I and bilateral sagittal split osteotomy (BSSO) using a surgery-first approach by a single team of surgeons using either the one-splint or two-splint technique with 3D surgical planning, surgical planning and post-surgical orthodontic treatment performed by a single orthodontist (L.Y-F), availability of patient-reported questionnaires completed before surgery (pre-treatment) and after orthodontic debonding (post-treatment), availability of digital photographs taken before orthodontic and OGS treatment (pre-treatment) and after orthodontic debonding (post-treatment) and the absence of congenital craniofacial anomalies. The study was conducted in accordance with the Helsinki Declaration [22] and reported according to the Strengthening the Reporting of Observational Studies in Epidemiology (STROBE) guidelines [23].

### Surgical planning

3D virtual orthognathic planning was done using Dolphin Imaging (Patterson Dental Supply, Winsconsin, USA) by the treating orthodontist based on CBCT, photographs and dental casts taken 1 month before OGS. The laboratory procedures were described in Liao et al. [16] and planning principles detailed in Liao et al. [24]. Surgical movements were planned to achieve hard and soft tissue symmetry based on individual diagnosis and clinical examination. The surgical splints were virtually designed and printed with 3D rapid prototyping using Objet30 OrthoDesk (Stratasys Ltd. Minnesota, USA).

### One-splint surgical technique

**Fig. 1:**
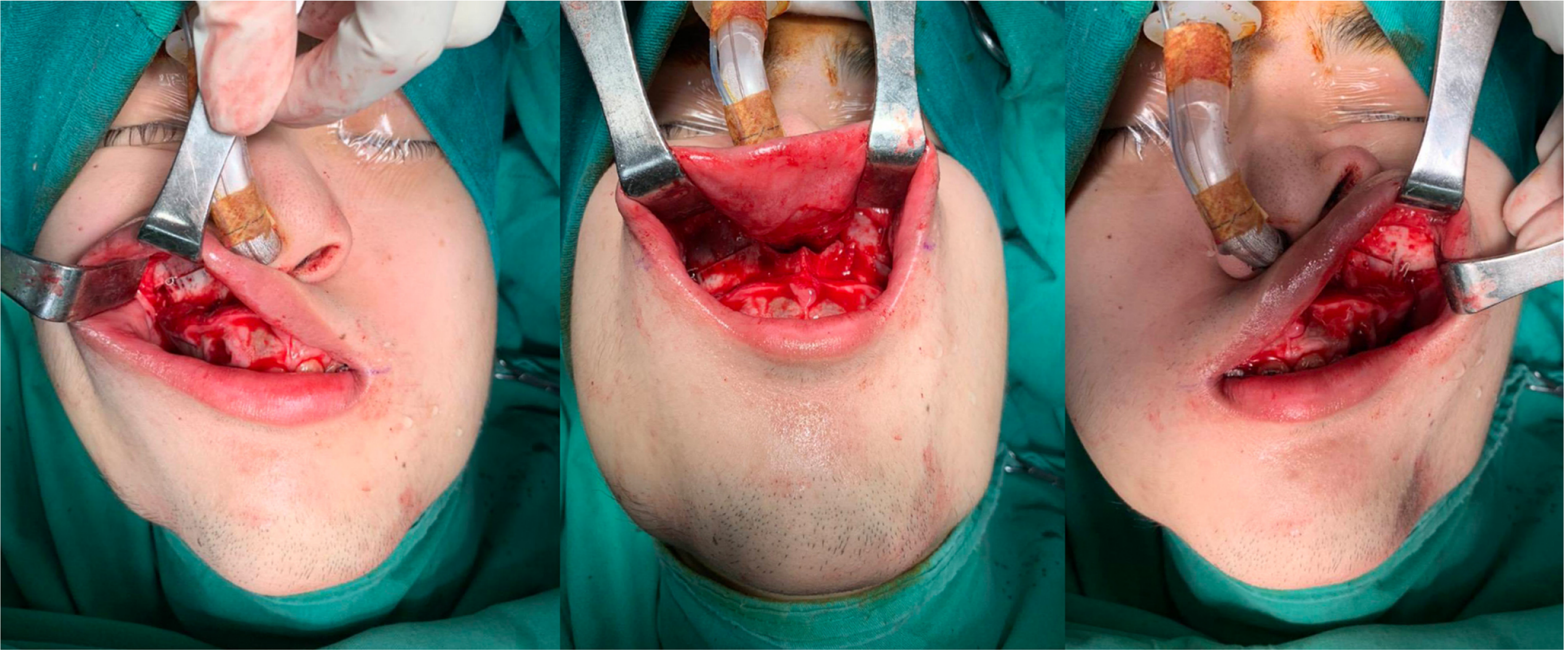
One-splint technique **—** wire fixation used to suspend the MMC whilst making intra-operative measurements

**Fig. 2:**
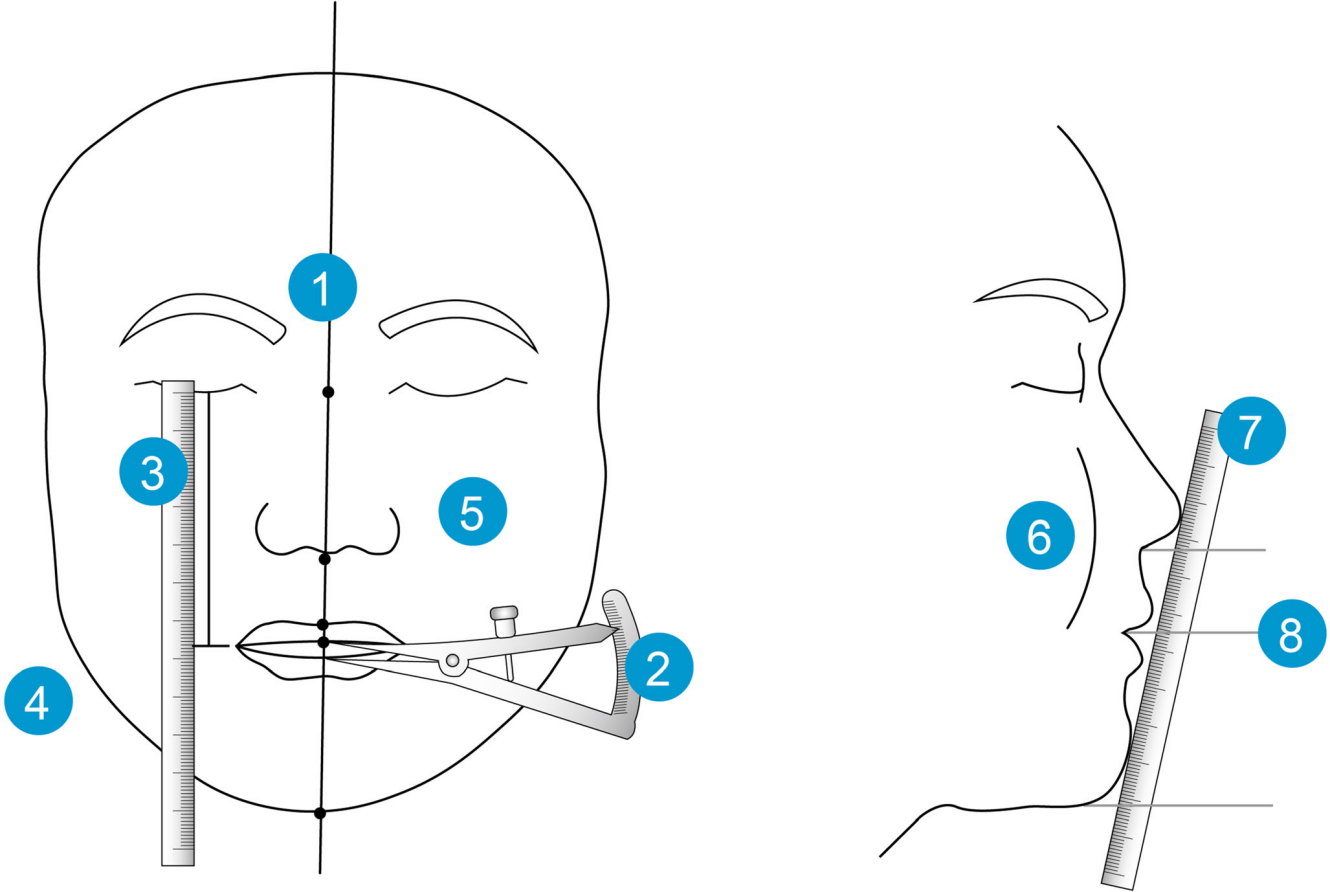
One-splint technique — schematic of intra-operative checkpoints: (1) midline coordination, (2) upper incisor show, (3) intercommissural line, (4) frontal contour symmetry, (5) frontal cheek symmetry, (6) paranasal fullness, (7) Ricketts E-line, and (8) lower face proportions

The MMC was completely mobilised with a LeFort I osteotomy and a BSSO, with or without segmental osteotomies. It was then put into intermaxillary fixation with the final occlusal splint. The MMC was repositioned based on the 3D plan, and metal wires on the best bone contact sites of the right and left maxilla were used to temporarily fix the MMC to the stable upper maxillary base (Fig. 1), with the metal wire suspension accommodating small adjustments of the maxillomandibular complex position based on the following intra-operative checkpoints: (1) midline coordination, (2) upper incisor show, (3) intercommissural line, (4) frontal contour symmetry, (5) frontal cheek symmetry, (6) paranasal fullness, (7) Ricketts E-line and (8) lower face proportions (Fig. 2).

1. Midline coordination was done from a true antero-posterior view. The soft tissue glabella, subnasale, midline of Cupid’s bow and chin point were marked with ink and aligned, together with the maxillary inter-incisive line, onto the facial midline decided by 3D virtual plan. When all the above landmarks could not be aligned perfectly, the alignment of the maxillary inter-incisive line with the midline of Cupid’s bow was prioritised. The remaining landmarks were lined up as best as possible by altering the MMC position. The lateral displacement of the nose due to nasal intubation was taken into account when performing midline coordination. The need for chin point correction with genioplasty was also assessed.
2. The upper incisor show was measured and adjusted to between 2 and 3 mm. Incisive show was modified by changing the amount of maxillary anterior impaction or extrusion and the amount of maxillary setback or advancement. Care was taken on this step as the nose is often displaced superiorly due to nasal intubation and maxillary exposure.
3. The distances from the left and right lip commissure to the corresponding lower eyelid were measured to be consistent with 3D virtual plan. In the absence of an eye cant, the distances should be similar on both sides. Altering the MMC roll by burring or grafting the zygomaticomaxillary buttress was done to change the position of the corresponding commissure and obtain better symmetry.
4. Frontal facial contours were visually assessed from a true antero-posterior view and were altered by modifying the MMC yaw, with the axis of rotation being the anterior maxillary midline. Need for genioplasty or mandibular contouring to improve contour symmetry was assessed at this time.
5. Frontal cheek fullness should be symmetrical and was altered by modifying the MMC yaw.
6. Laterally, paranasal fullness or depression was assessed and adjusted by modifying the antero-posterior position and pitch of the MMC.
7. Laterally, the need for advancement or setback genioplasty was confirmed by verifying the Ricketts E line with a ruler.
8. The classic lower face proportions (subnasale to intercommissural plane, one-third; intercommissural plane to chin point, two-thirds) were checked to assess the need for chin elongation or shortening with genioplasty.

**Fig. 3:**
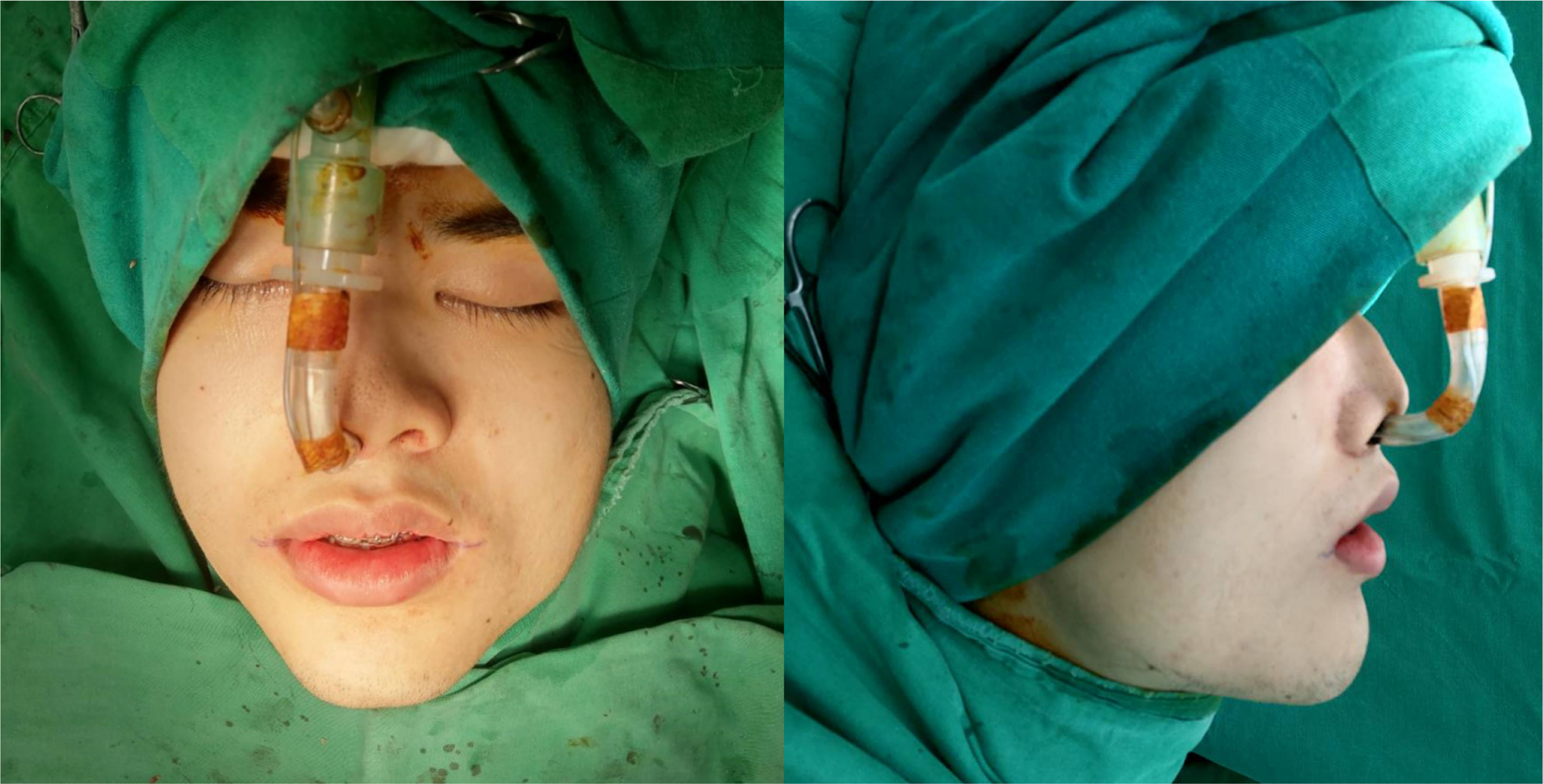
One-splint technique **—** intra-operative view for making measurements and verifying checkpoints

After the MMC position was confirmed, titanium bone plates and screws were used for rigid fixation and placed at the maxilla first, followed by the mandible. Genioplasty or mandibular contouring was performed as needed based on the above intra-operative assessments, and all checkpoints were re-verified (Fig. 3).

### Two-splint surgical technique

**Fig. 4:**
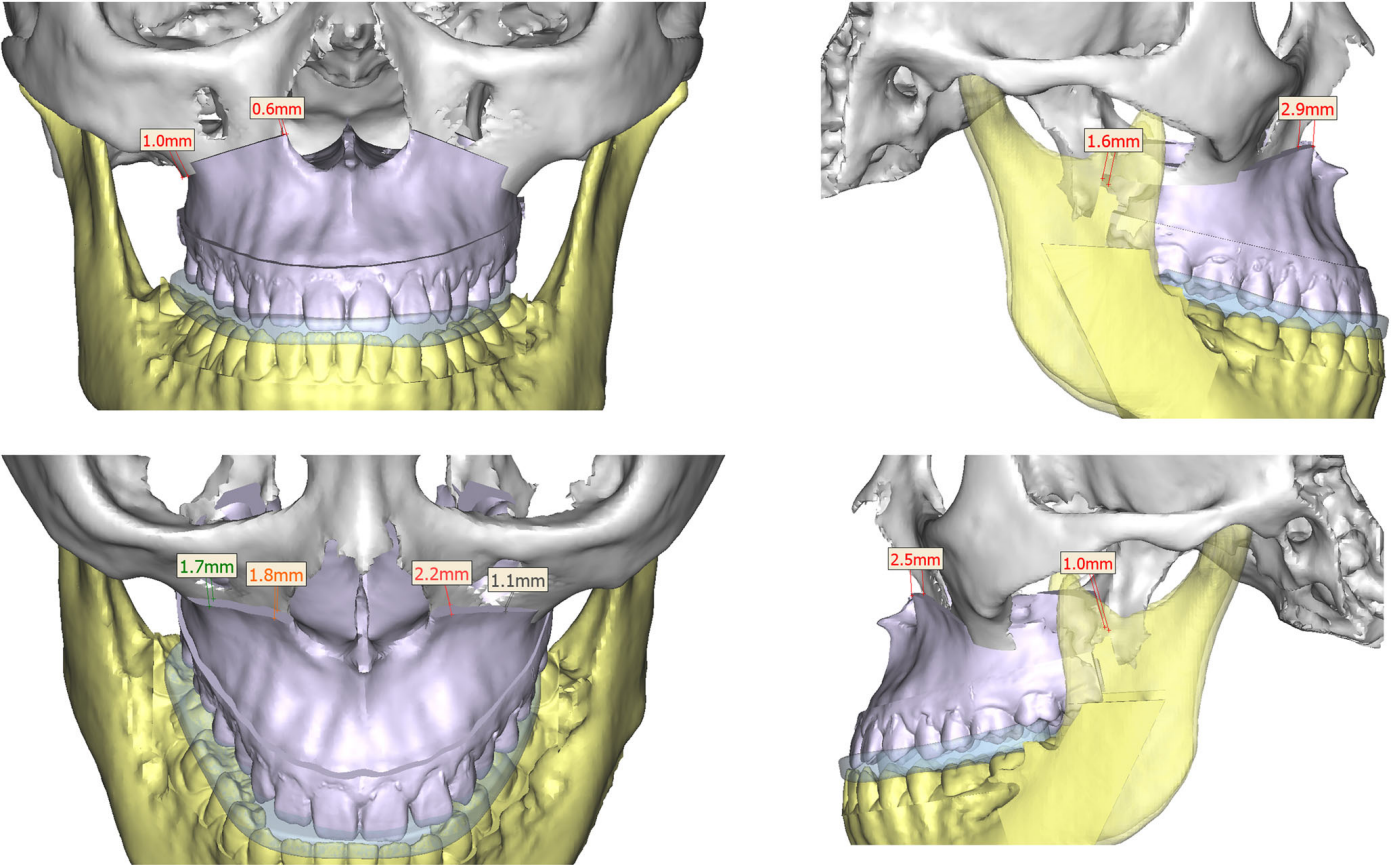
Two-splint technique **—** example of a virtual surgical plan of maxillary movement with the intermediate splint in place

**Fig. 5:**
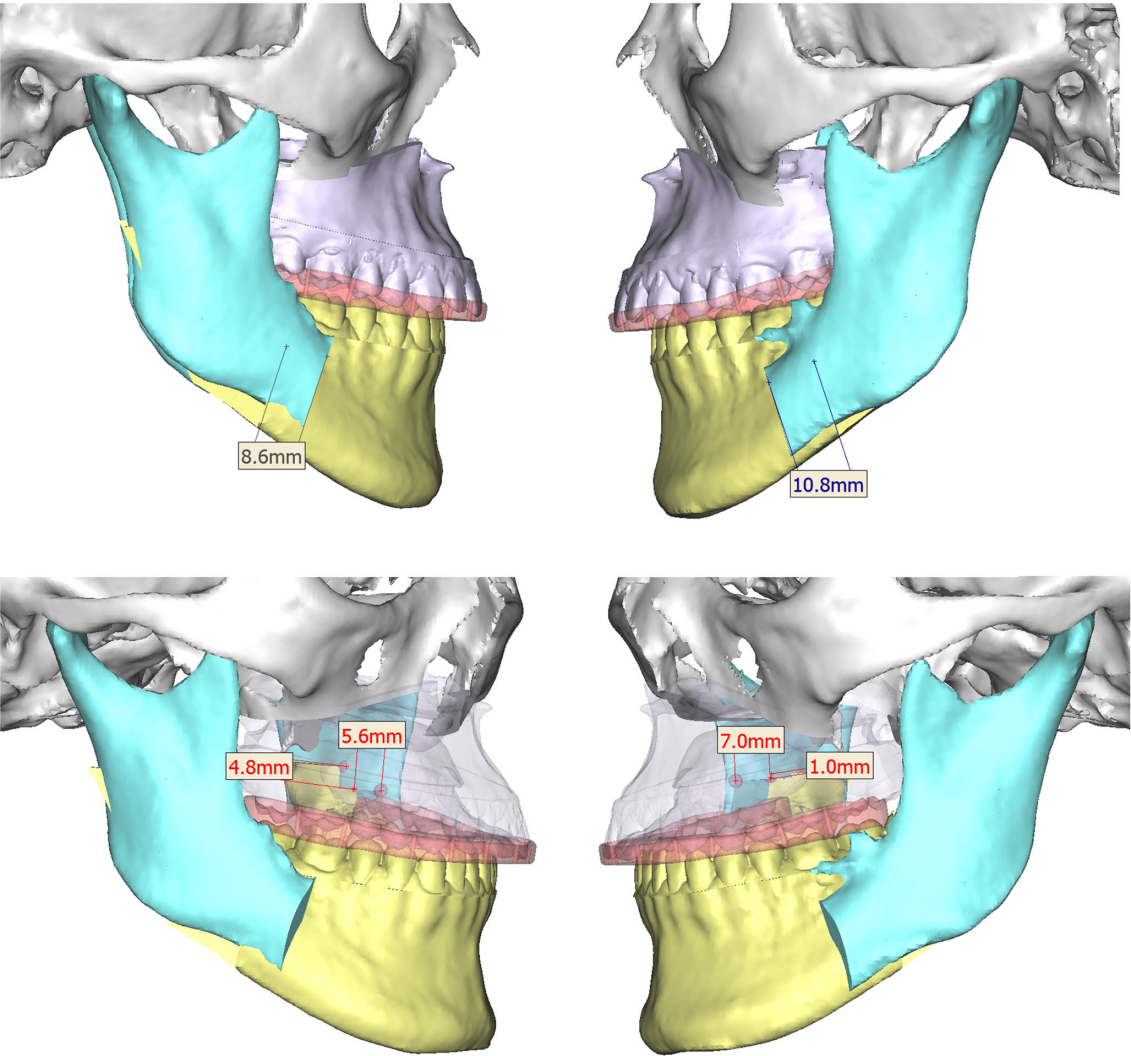
Two-splint technique **—** example of a virtual surgical plan of mandibular movement with the final splint in place

All patients in the two-splint technique group underwent a maxilla-first sequence. Pre-surgically, an additional bite registration with 3–8 mm bite opening was taken in supine position to obtain an open-mouth maxillomandibular registration and used to reduce inaccuracies in mandibular autorotation and eliminate interferences in the intermediate surgical splint. LeFort I osteotomy with or without segmental osteotomy was used to mobilise the maxilla. Intermediate splint was used with intermaxillary wiring to form the intermediate MMC (Fig. 4), which moves according to bilateral temporomandibular joints and guides the position of maxilla. The vertical position of maxilla was adjusted according to the planned movements, after which titanium bone plates and screws were used for rigid fixation of the intermediate MMC. Intermaxillary wiring was released, and BSSO was done on the mandible and positioned using the final splint (Fig. 5) before the final mandible position was confirmed. Titanium bone plates and screws were used for rigid fixation of the mandible. Genioplasty or mandibular contouring was performed as needed based on intra-operative assessment of soft-tissue profile, proportion and symmetry.

### Soft tissue analysis

Frontal photographs were taken pre-treatment and post-treatment. A professional photographer used a Canon EOS 350D digital camera (Canon, Inc., Tokyo, Japan), with the resolution set at 2496 × 1664 pixels to photograph patients according to the photographic standards of the European Association for Cranio-Maxillo-Facial Surgery [25, 26].

**Fig. 6:**
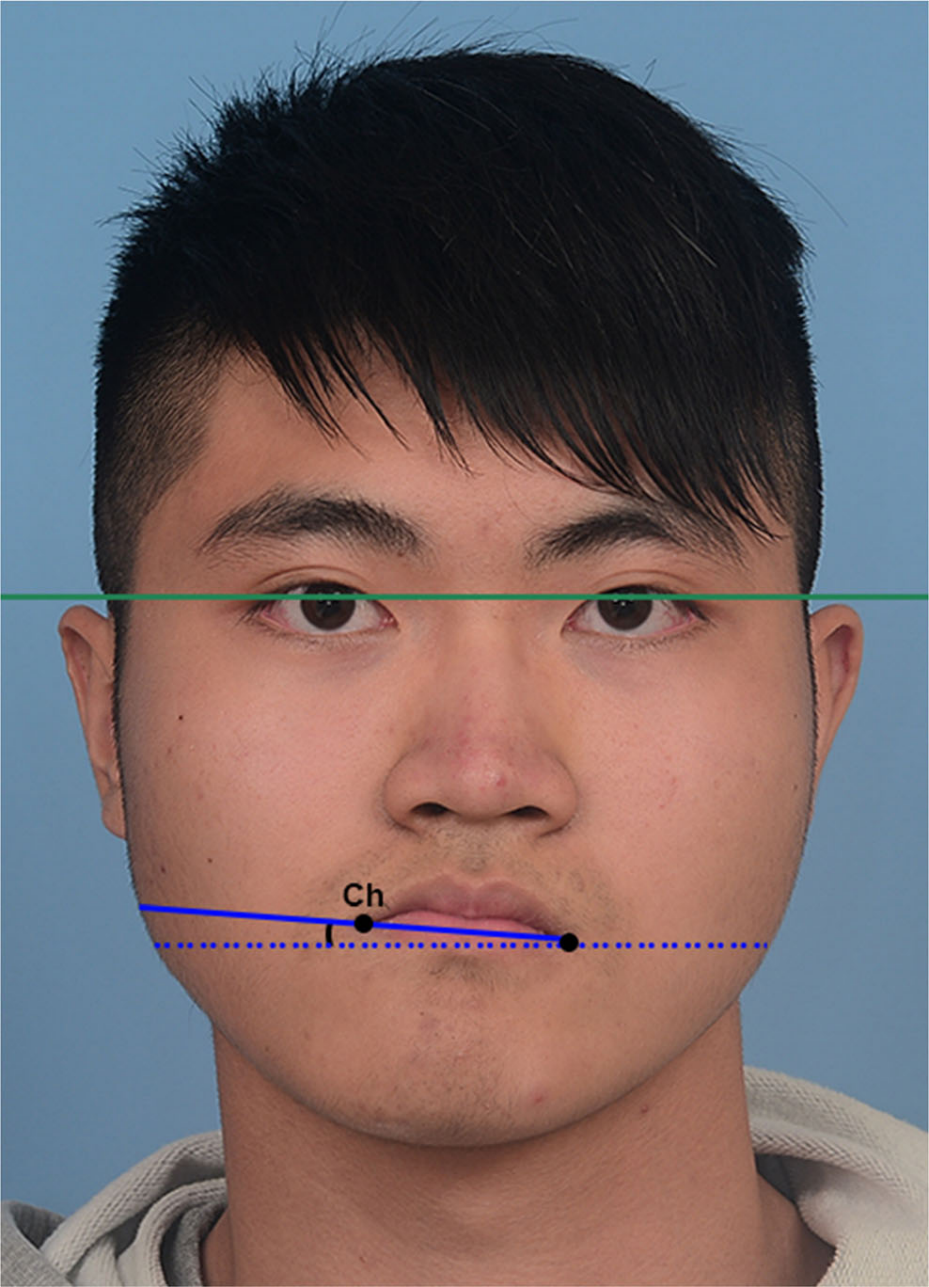
Intercommissural line deviation. The interpupillary line is shown in green. Ch: Cheilion. Intercommissural line deviation: angle formed between the intercommissural line (blue, Ch-Ch) and the interpupillary line (green)

**Fig. 7:**
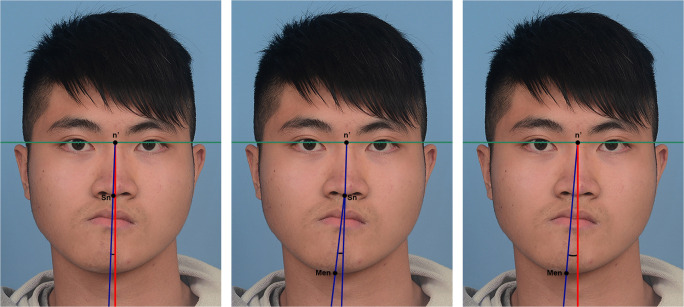
Representative images of Facial Midline Symmetry Index measurements. The interpupillary line is shown in green. n’: Nasion projection on the interpupillary line. Sn: subnasale. Men: menton. (Left) Midface deviation: angle formed between the midface deviation line (blue line, n’-Sn) and facial midline (red line, perpendicular to interpupillary line). (Centre) Chin from midface deviation: angle formed between the midface deviation line (blue line, n’-Sn) and Sn-Men (blue line). (Right) Chin deviation: angle between n’-Men (blue line) and facial midline (red line)

**Fig. 8:**
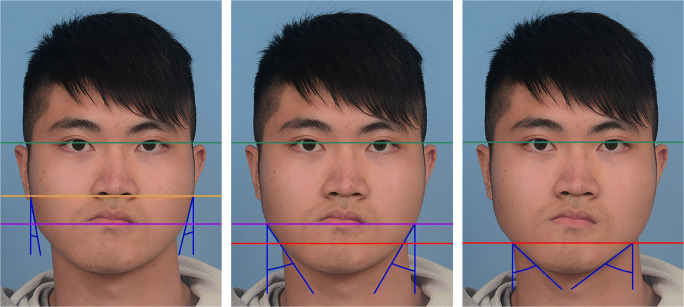
Representative images of Facial Contour Symmetry Index measurements. The interpupillary line is shown in green. (Left) Upper contour deviation: absolute difference between the right and left upper contour angle, which is the angle between the tangent line from the upper contour to the facial midline (blue lines). (Centre) Middle contour deviation: absolute difference between the right and left middle contour angle, which is the angle between the tangent line from the middle contour to the facial midline (blue lines). (Right) Lower contour deviation: absolute difference between the right and left lower contour angle, which is the angle between the tangent line from the lower contour to the facial midline (blue line)

Seven angular anthropometric measurements were made on the digital photographs (Figs. 6, 7 and 8). These were performed with Photoshop 9.0 (Adobe Systems, Inc., San Jose, CA, USA) by an experienced researcher. The seven measurements constituted the Facial Symmetry Index, which is adapted from the Facial Midline Symmetry Index [7] and has been validated in previous studies [15, 16]. The index comprises one lip cant measurement (Fig. 6), three midline facial measurements (Fig. 7) and three facial contour measurements (Fig. 8). The three midline measurements are combined into a Facial Midline Symmetry Index, whilst the three contour measurements are combined into a Facial Contour Symmetry Index. All seven measurements are combined to calculate an Overall Facial Symmetry Index. The higher the value of individual measurements and overall indices, the greater the degree of asymmetry, with zero representing perfect symmetry.

The same researcher measured 10 photographs randomly selected from a collection of pre-surgery images (*n* = 5) and post-surgery images (*n* = 5) to assess intra-observer reliability. The intraclass correlation coefficient suggested excellent reliability (*p* < .05; range = 0.986 to 0.996).

### Patient-reported questionnaire

Pre-surgery and post-debonding, all patients completed the Orthognathic Quality of Life Questionnaire (OQLQ). OQLQ consists of four domains: Facial Esthetics, Oral Function, Awareness of Dentofacial Esthetics and Social Aspects of Dentofacial Deformity, and a total score ranging from 0 to 88. A lower score indicates better QoL [27, 28]. The Chinese version of OQLQ was used [29–31].

### Statistical analysis

Demographic data, Facial Symmetry Index and OQLQ scores were analysed with descriptive statistics. Measurements between groups were compared with independent *t*-tests and chi-squared tests where indicated. Before-and-after treatment measurements were compared with paired *t*-tests. All tests were two-tailed; statistical significance was set at *p* < 0.05. The Statistical Package for Social Sciences was used (Version 21.0; SPSS Inc., Chicago, Illinois, USA).

## Results

### Patients

Eighty patients met the selection criteria. There were 34 patients in the one-splint group and 46 patients in the two-splint group (Table 1). The most common accessory surgical procedure was genioplasty. The one-splint group had significantly more negative pre-treatment ANB.

**Table 1:** Clinical characteristics of one- and two-splint groups

Characteristics	One-splint (*n* = 34)	Two-splint (*n* = 46)	*p*-value
% Female (*n*)	47% (16)	54% (25)	0.519
Age at surgery (years) (mean ± standard deviation)	22 ± 5	25 ± 6	0.052
Initial ANB angle (degrees) (mean ± standard deviation)	− 4.9 ± 2.4	− 2.6 ± 2.2	< 0.001*
Initial overjet (mm) (mean ± standard deviation)	− 2.6 ± 2.6	− 2.2 ± 3.7	0.293
Initial overbite (mm) (mean ± standard deviation)	1.0 ± 2.1	0.6 ± 2.2	0.417
% With maxilla segmentation (*n*)	15% (5)	20% (9)	0.572
% With mandible segmentation (*n*) (Kole’s Osteotomy)	3% (1)	0% (0)	0.242
% With genioplasty (*n*)	53% (18)	74% (34)	0.052
% With mandible contouring (*n*)	24% (8)	15% (7)	0.346

*Statistically significant *p*-values

### Symmetry

Both groups had significant improvement in intercommissural line deviation after treatment (Table 2). The two-splint group had significant improvement in all midline symmetry measures after treatment. The one-splint group showed no significant improvement in midface deviation but had significant improvement in the remaining midline symmetry measures including the combined Facial Midline Symmetry Index. The two-splint group had significant improvement in all contour symmetry measures after treatment. The one-splint group had significant improvement in middle and lower contour deviation but no significant change in upper contour deviation and the combined Facial Contour Symmetry Index. Both groups had significant improvement in the combined Overall Facial Symmetry Index after treatment.

**Table 2:** Facial symmetry index of one- and two-splint groups before and after treatment

Measurement (higher score indicates greater asymmetry)	One-splint (*n* = 34)			Two-splint (*n* = 46)			One- vs two-splint	
	Pre-treatment (mean ± standard deviation)	Post-treatment (mean ± standard deviation)	*p*-value	Pre-treatment (mean ± standard deviation)	Post-treatment (mean ± standard deviation)	*p*-value	Pre-treatment *p*-value	Post-treatment *p*-value
Intercommissural line deviation (degrees)	2.0 ± 1.4	1.1 ± 0.9	0.001*	2.5 ± 1.5	1.1 ± 1.0	< 0.001*	0.131	0.761
Midface deviation (degrees)	0.5 ± 0.8	0.4 ± 0.6	0.162	0.6 ± 0.9	0.4 ± 0.7	0.020*	0.793	0.843
Chin from midface deviation (degrees)	5.6 ± 2.8	1.8 ± 1.6	< 0.001*	5.5 ± 2.6	1.5 ± 1.6	< 0.001*	0.855	0.412
Chin deviation (degrees)	3.8 ± 1.9	1.2 ± 0.9	< 0.001*	3.7 ± 1.4	1.1 ± 1.0	< 0.001*	0.876	0.567
Facial midline symmetry index	9.9 ± 4.8	3.4 ± 2.5	< 0.001*	9.7 ± 4.0	3.0 ± 2.6	< 0.001*	0.869	0.501
Upper contour deviation (degrees)	3.4 ± 3.1	3.2 ± 2.5	0.550	3.9 ± 2.8	2.5 ± 1.6	0.013*	0.430	0.216
Middle contour deviation (degrees)	3.3 ± 2.3	2.8 ± 4.3	0.041*	3.8 ± 2.4	2.1 ± 1.5	< 0.001*	0.378	0.347
Lower contour deviation (degrees)	4.7 ± 4.3	3.1 ± 2.7	0.011*	4.3 ± 3.6	2.8 ± 2.2	0.020*	0.584	0.630
Facial contour symmetry Index	11.5 ± 7.0	9.1 ± 6.3	0.078	12.0 ± 6.4	7.4 ± 3.6	< 0.001*	0.654	0.138
Overall facial symmetry index	23.3 ± 11.7	13.6 ± 7.3	< 0.001*	24.2 ± 10.3	11.5 ± 5.0	< 0.001*	0.658	0.123

*Statistically significant *p*-values

Comparing the two groups, there were no differences in all the individual and combined symmetry measurements between the groups pre- or post-treatment.

### Quality of life

Both groups had significant improvement in all OQLQ domains and total scores after treatment (Table 3).

**Table 3:** OQLQ of one- and two-splint groups before and after treatment

Measurement (higher score indicates poorer QoL)	One-splint (*n* = 34)			Two-splint (*n* = 46)			One- vs two-splint	
	Pre-treatment (mean ± standard deviation)	Post-treatment (mean ± standard deviation)	*p*-value	Pre-treatment (mean ± standard deviation)	Post-treatment (mean ± standard deviation)	*p*-value	Pre-treatment *p*-value	Post-treatment *p*-value
Facial Esthetics (0 to 20)	14.1 ± 3.2	6.2 ± 4.0	< 0.001*	12.9 ± 4.5	6.3 ± 3.4	< 0.001*	0.183	0.924
Oral Function (0 to 20)	12.7 ± 4.1	4.7 ± 2.9	< 0.001*	9.1 ± 4.5	4.7 ± 2.8	< 0.001*	0.001*	0.912
Awareness of Dentofacial Esthetics (0 to 16)	10.8 ± 3.1	5.3 ± 3.1	< 0.001*	7.9 ± 3.9	4.6 ± 2.7	< 0.001*	0.001*	0.316
Social Aspect of Dentofacial Deformity (0 to 32)	20.9 ± 6.9	7.6 ± 6.8	< 0.001*	15.2 ± 8.5	7.8 ± 5.8	< 0.001*	0.002*	0.689
OQLQ Total (0 to 88)	58.4 ± 13.5	23.7 ± 15.1	< 0.001*	45.1 ± 19.3	23.3 ± 12.1	< 0.001*	0.001*	0.902

*Statistically significant *p*-values

Comparing the two groups, all pre-treatment OQLQ domains and total scores except for the Facial Esthetics domain were significantly higher in the one-splint group than the two-splint group. These differences were resolved after treatment, with no significant differences in post-treatment OQLQ domains and total scores between the two groups.

## Discussion

To reduce risk of bias, there was complete inclusion of all patients meeting the study criteria. However, the groups were not equivalent at baseline, differing in sample size, ANB and OQLQ scores. The larger number of two-splint patients reflects the difference in the number of recruitment years, with the two-splint group being recruited from 3 years of clinical cases (2017–2019) and the one-splint group being drawn from 2 years (2015–2016). Due to general adoption of the two-splint technique in the Center, contemporaneous study groups were not achievable.

The more negative ANB angle in the one-splint group suggests that the one-splint group had more severe skeletal Class III discrepancy than the two-splint group. However, despite the difference in skeletal deformity in the antero-posterior plane, the degree of frontal soft tissue asymmetry measured by the Facial Symmetry Index was not significantly different between the two groups before treatment. All OQLQ domains and total scores except the Facial Esthetics domain were significantly higher in the one-splint group before treatment, suggesting a significantly worse QoL in the one-splint group compared to the two-splint group. This was likely due to the pre-treatment difference in ANB. OQLQ scores have been found to be significantly negatively correlated to ANB in Japanese Class III OGS patients, suggesting QoL is adversely affected by the degree of skeletal discrepancy [32]. Hence, due to the study design, there were differences between the study groups in terms of the sample size, severity of skeletal deformity and the level of impairment on QoL. An additional limitation was the lack of homogeneity in surgical procedures done within the two groups, with a percentage of patients in each group requiring segmental osteotomies, genioplasty or mandible contouring. However, there were no significant differences in the percentage of segmental osteotomies, genioplasty or mandible contouring between the two study groups. Any interpretations of the results must be coloured by these limitations.

Compared to 2D imaging, 3D imaging provides better diagnosis of facial asymmetry, surgical planning and treatment transfer. Because of this, hard tissue symmetry is more easily achieved with 3D surgical planning compared with 2D planning and can largely be considered a solved problem [13, 14, 17, 18]. Unfortunately, acceptable hard tissue symmetry does not necessarily translate into good soft tissue symmetry, and patients with symmetric hard tissues may still have asymmetric soft tissues [7, 9, 33]. A recent study of Class III facial asymmetry patients found that despite bimaxillary OGS, soft tissue asymmetry did not change significantly after treatment and throughout 24-month follow-up [34]. Despite this, OGS literature is still largely focused on evaluating hard tissue metrics and execution of hard tissue plans, with significantly less attention on soft tissue outcomes [13, 14, 17, 35, 36]. In addition, the focus of most asymmetry studies is on midline structures and the deviation from the mid-sagittal plane [13, 14, 17, 37, 38]. However, patients seeking OGS for asymmetry can present with significant contour asymmetry.

Whilst accurate execution of the surgical plan and the resulting hard tissue midline symmetry are necessary outcome metrics, they are insufficient to define surgical success [16]. Patients request OGS not only to remedy deformities and functional problems but also to enhance their body image and QoL and reduce perceived social stigma. OGS may help to enhance self-perceived attractiveness and self-confidence of individuals [39]. Frontal soft tissue symmetry is the only metric that is easily assessed visually by patients and in practice is likely inspected daily in the mirror [16, 40]. The patient’s perceived frontal improvement translates into patient satisfaction and psychosocial benefits [16, 18, 39]. For this reason, this study focused on frontal soft tissue outcomes, including soft tissue contour, as well as patient-reported QoL.

In OGS, the two-splint technique is classically considered the gold standard for surgical plan transference, whereas one-splint technique is controversial [5, 8]. The addition of 3D planning to one-splint technique has improved visualisation of yaw asymmetry [14] and enhanced hard and soft tissue results in asymmetry patients [13–16]. Nonetheless, with the one-splint technique, there was no precise way of translating the plan to the surgical field, with significant deviations from the 3D plan seen [13, 14]. It was hypothesised that with more accurate implementation of the 3D surgical plan, the two-splint technique might produce better soft tissue symmetry.

This study found that the two-splint technique may be better able to improve contour symmetry than the one-splint technique, as the two-splint technique was able to improve all symmetry measurements, whereas the one-splint technique produced no significant improvement in Midface Deviation, Upper Contour Deviation measurements and the combined Facial Contour Symmetry Index. However, both the one-splint and two-splint techniques significantly improved all other symmetry measurements as well as the combined Facial Midline Symmetry Index and Overall Facial Symmetry Index. Furthermore, there were no significant differences in any facial symmetry measures after treatment between the one-splint and two-splint groups.

This lack of significant difference in final outcomes can be explained by the fact that the one-splint technique was developed specifically for frontal soft tissue asymmetry [6, 7, 41]. The intra-operative checkpoints emphasised in the one-splint technique were designed based on measures used in the Facial Midline Symmetry Index [7]. The focus on midline structures may have also unduly influenced the results of the Overall Facial Symmetry Index. The one-splint technique was found to produce adequate frontal soft tissue symmetry in patients [7, 15, 16]. Although the one-splint technique produces significant error in MMC pitch compared to the 3D surgical plan [13], inaccuracies in hard tissue pitch may manifest more in the lateral profile and less in frontal soft tissue symmetry.

One previous study compared the one-splint, free-hand MMC positioning technique against a guided maxilla positioning technique [18]. Compared with patients who had 2D surgical planning and one-splint technique OGS, patients who had 3D surgical planning and printed extra-skeletal MMC positioning guides showed both better hard tissue symmetry as well as better patient satisfaction after OGS. Although the study did not use an intermediate splint to position the maxilla, the findings suggest that guided MMC positioning has superior results over free-hand MMC positioning. However, the fact that the one-splint group used 2D planning whilst the guided MMC positioning group used 3D planning makes it difficult to draw firm conclusions, as 3D planning with one-splint technique has shown good results in asymmetric patients [13–16].

With regard to QoL, this study found that both the one-splint and the two-splint groups experienced significant improvement in OQLQ scores after treatment, which is similar to other studies on East Asian Class III OGS patients [30–32]. In addition, despite the worse QoL in the one-splint group pre-treatment, there was no significant difference between the two groups in OQLQ post-treatment, suggesting a similar level of QoL was attained. However, a direct comparison of QoL changes between the two groups may not be meaningful due to the significantly worse pre-treatment QoL in the one-splint group.

Finally, the authors’ Craniofacial Center has been using the surgery-first approach for more than 30 years and has an established surgery-first practice and clinical protocol. However, surgical outcomes of the surgery-first technique can be variable [42], and the results of this study may not be generalizable to surgeons and orthodontists who have limited experience in the surgery-first approach.

To the best of the authors’ knowledge, this is the first study that compares the 3D planned one-splint technique with the 3D planned two-splint technique. The results suggest that the two-splint technique may better improve contour symmetry, but there are no significant differences in overall soft tissue symmetry and in QoL outcomes between the two techniques. However, although frontal soft tissue and post-treatment QoL outcomes are similar, two-splint technique has intra-operative benefits over one-splint as it is also less surgically demanding, has less of a learning curve and depends less heavily on surgeon experience [7, 12]. A simpler and less demanding surgery may reduce operative time, intra-operative blood loss and post-operative hospital stay [43, 44]. This might lead to better patient experience and higher patient satisfaction immediately post-operatively. This study did not investigate these parameters, and future work should investigate these questions.

## Conclusions

OGS treatment with both the one-splint and two-splint techniques significantly improves soft tissue symmetry and QoL in skeletal Class III asymmetry patients. Two-splint technique may better improve contour symmetry. There were no differences in soft tissue symmetry and QoL after OGS treatment in skeletal Class III asymmetry patients treated with either the one-splint or two-splint technique.

## Supplementary Information

ESM 1 (DOC 87 kb)
